# Supplementary material for: Chromosomal variations of Lycoris species revealed by FISH with rDNAs and centromeric histone H3 variant associated DNAs
Source: PLoS One. 2021 Sep 30;16(9):e0258028. doi: 10.1371/journal.pone.0258028 (PMC8483392; doi:10.1371/journal.pone.0258028)
Supplement: S1 Fig — Crude nuclear proteins (lanes 1, 4 and 7), LaCENH3 antibody immunoprecipitated proteins (lanes 2, 5 and 8) and H3K4me3 antibody immunoprecipitated proteins (lanes 3, 6 and 9) were separated on a polyacrylamide gel, transferred onto a PVDF membrane and detected with pre-serum, LaCENH3 and H3K4me3 antibodies, respectively. Pre-serum was used as a negative control. The immunoprecipitated proteins were obtained from equal amount of nuclear proteins. The protein loading volume was normalized to equal amount of crude nuclear proteins. The relative molecular weight of protein was indicated at the left. (PDF) [file pone.0258028.s001.pdf]

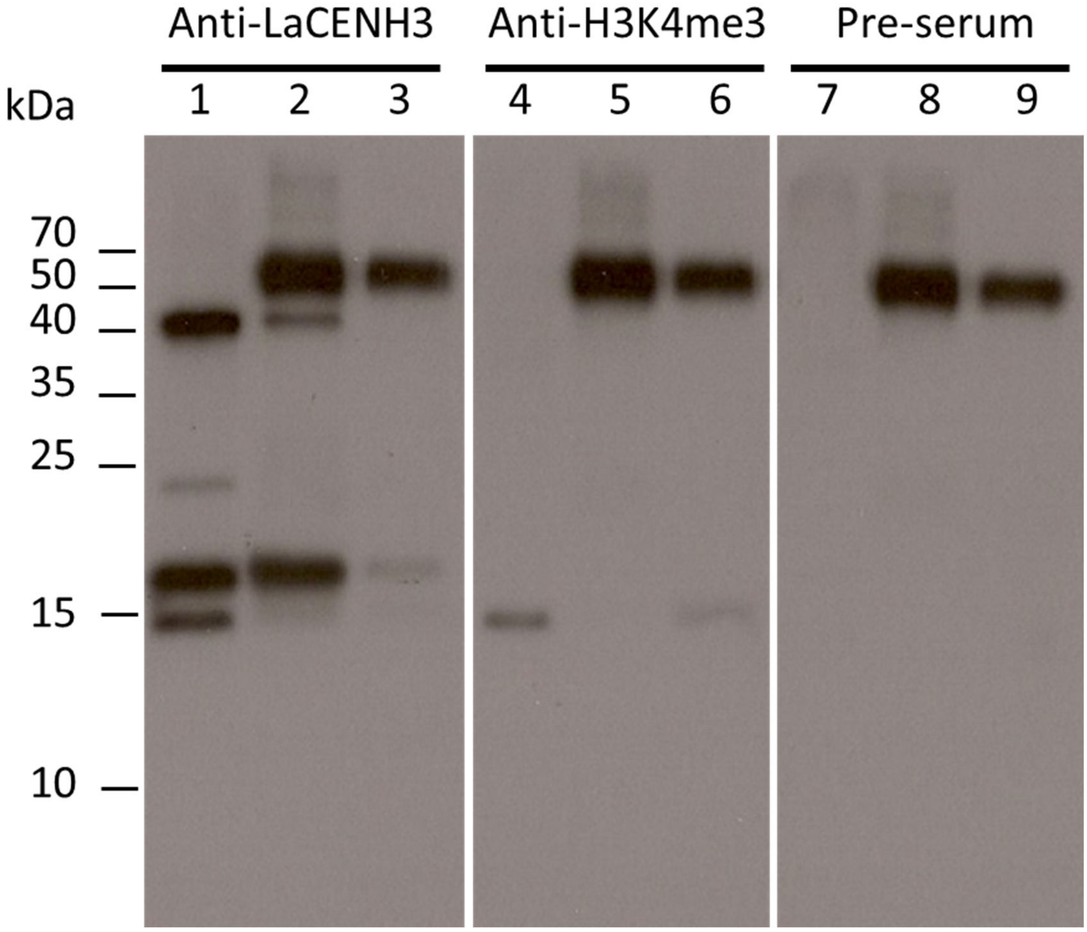

S1_Fig. Western blot analysis of LaCENH3 and histone 3 proteins.

Crude nuclear proteins (lanes 1, 4 and 7), LaCENH3 antibody immunoprecipitated proteins (lanes 2, 5 and 8) and H3K4me3 antibody immunoprecipitated proteins (lanes 3, 6 and 9) were separated on a polyacrylamide gel, transferred onto a PVDF membrane and detected with pre-serum, LaCENH3 and H3K4me3 antibodies, respectively. Pre-serum was used as a negative control. The immunoprecipitated proteins were obtained from equal amount of nuclear proteins. The protein loading volume was normalized to equal amount of crude nuclear proteins. The relative molecular weight of protein was indicated at the left.
