# Supplementary material for: Chromosomal variations of Lycoris species revealed by FISH with rDNAs and centromeric histone H3 variant associated DNAs
Source: PLoS One. 2021 Sep 30;16(9):e0258028. doi: 10.1371/journal.pone.0258028 (PMC8483392; doi:10.1371/journal.pone.0258028)
Supplement: S2 Fig — (Scale bar = 10 μm). (PDF) [file pone.0258028.s002.pdf]

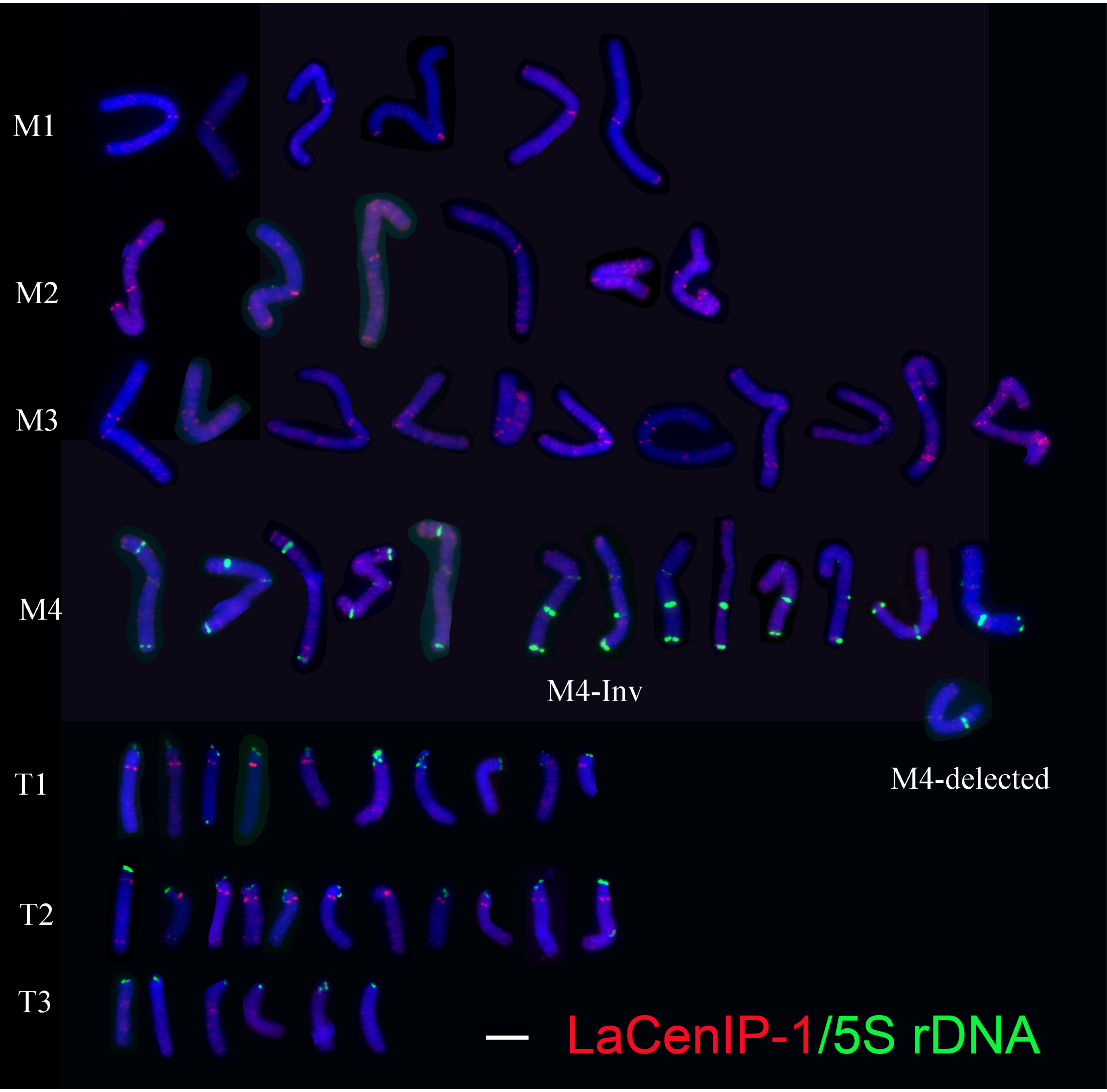
M1
M2
M3
M4
M4-Inv
M4-delected
T1
T2
T3
LaCenIP-1/5S rDNA

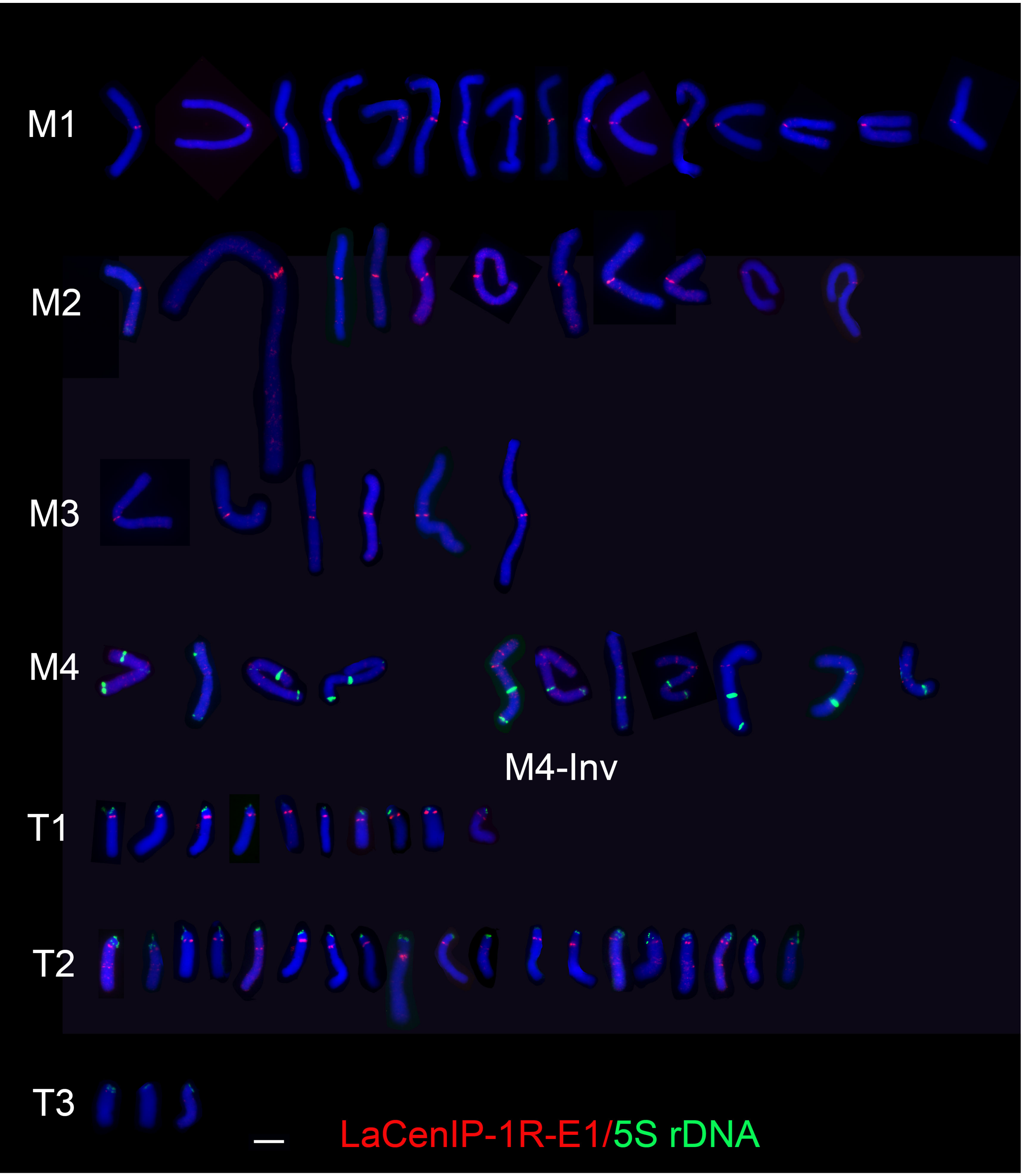


**S2_Fig.** Collection of chromosomes from several FISH experiments with LaCenIP-1 and LaCenIP-1R-E1 as probes showing unique distribution patterns consistently detected on individual chromosomes. (Scale bar = 10 μm)
